# The Relationship of Anxiety and Stress With Working Memory Performance in a Large Non-depressed Sample

**DOI:** 10.3389/fpsyg.2019.00004

**Published:** 2019-01-23

**Authors:** Karolina M. Lukasik, Otto Waris, Anna Soveri, Minna Lehtonen, Matti Laine

**Affiliations:** ^1^Department of Psychology, Åbo Akademi University, Turku, Finland; ^2^Department of Psychology and Logopedics, Faculty of Medicine, University of Helsinki, Helsinki, Finland; ^3^Center for Multilingualism in Society Across the Lifespan, Department of Linguistics and Scandinavian Studies, University of Oslo, Oslo, Norway; ^4^Turku Brain and Mind Center, University of Turku, Turku, Finland

**Keywords:** anxiety, stress, working memory, cognition, healthy adults

## Abstract

Clinical anxiety and acute stress caused by major life events have well-documented detrimental effects on cognitive processes, such as working memory (WM). However, less is known about the relationships of state anxiety or everyday stress with WM performance in non-clinical populations. We investigated the associations between these two factors and three WM composites (verbal WM, visuospatial WM, and n-back updating performance) in a large online sample of non-depressed US American adults. We found a trend for a negative association between WM performance and anxiety, but not with stress. Thus, WM performance appears rather robust against normal variation in anxiety and everyday stress.

## Introduction

Cognitive performance can be affected by a number of factors, including non-cognitive ones like the emotional state of the test-taker (Gray, 2001; Owens et al., 2012; Storbeck, 2012; Luck and Vogel, 2013). In the emotional sphere, major factors that can affect demanding cognitive performance include stress and anxiety. While more pronounced symptoms on each of these two factors are clearly linked to impaired cognitive performance (Sandi, 2013; Maloney et al., 2014; Moran, 2016), their effects are less clear when testing cognition in non-clinical populations. However, for both theoretical and practical purposes, it is essential to know whether even normal variability in stress and anxiety has an impact on cognition. This is relevant also for working memory (WM) that represents a core cognitive function. It is a limited-capacity temporary memory storage system that is constantly updated (Baddeley, 2003). It serves as a mental platform for ongoing activities, being crucial for purposeful behavior and flexible interaction with the environment. WM is an object of extensive study both for basic research and for clinical assessment, and it is thus important to clarify factors that affect WM performance. In the present study, we examined the relationships between WM performance and stress and anxiety in a large non-depressed adult sample.

### Working Memory and Anxiety

Anxiety is a state of heightened vigilance (Grillon, 2002) that is associated with an increase in overall sensory sensitivity due to uncertainty or conflict (Gray, 2001; Cornwell et al., 2007; Eysenck et al., 2007; Grupe and Nitschke, 2013). A characteristic feature of anxiety is the limited control over worrying thoughts and attentional biases, contributing to a greater focus on negative stimuli (Matthews and Wells, 1996). It has been shown that anxiety disrupts cognitive performance (Maloney et al., 2014), including WM (Moran, 2016). This relationship works both ways, as cognitive impairment can lead to increased anxiety (Petkus et al., 2017).

In this study, we focused on self-reported state anxiety, the immediate sensation of feeling anxious, rather than temporally stable trait anxiety. The attentional control theory, proposed by Eysenck et al. (2007), suggests that state anxiety impairs cognitive performance by giving greater influence to the stimulus-driven (bottom-up) attentional system. The greater the anxiety, the more disruption this causes. A later paper on attentional control theory suggests that anxiety might affect only the executive component of WM (Eysenck and Derakshan, 2011): in a dual-task study of anxiety, the primary WM task performance in high anxious individuals decreased only if the additional task required executive control (Eysenck et al., 2005; see also Christopher and MacDonald, 2005). A study by Gustavson and Miyake (2016) showed that worry is also associated with impaired WM updating.

A recent meta-analysis by Moran (2016) examined the relationship between anxiety and WM capacity. Based on 177 samples, this meta-analysis on correlative studies found a moderate but reliable association so that higher anxiety was related to lower WM performances (overall Hedge’s *g* = -0.334). This held across anxiety type (state, trait), sample type (clinical, non-clinical), WM task paradigm (simple span, complex span, n-back), and WM content (spatial, phonological, visual). These findings speak for a rather general relationship that could be fitted to the attentional control account. However, Moran (2016) highlights various limitations of this research, including reliance on single measures of WM that makes it impossible to separate task-specific and task-general effects. Thus far, there have been no studies examining the relationships between state anxiety and WM domains at latent variable level.

### WM and Stress

In terms of both emotional components and the underlying neurocircuitry, there is a significant overlap between stress and anxiety, but stress encompasses both avoidant (anxious) and proactive responses. In turn, fear and anxiety can be experienced even in the absence of the neuroendocrine cascade that is related to stress reaction (Miller and O’Callaghan, 2002), just as stress does not necessarily entail experiencing fear or anxiety (Shin and Liberzon, 2010). As regards cognitive effects, it appears that stress and anxiety behave in similar ways: it has been shown that under stress, controlled attention resources are reduced as they are allocated to the potential threat (Klein and Boals, 2001). However, the inverted-U theory of acute stress (Mizoguchi et al., 2000; Muse et al., 2003; Sandi, 2013; Sapolsky, 2015) states that this effect depends on stress levels related to the test situation: moderate stress may enhance cognitive performance, while both low (unmotivating) and high (overwhelming) stress are associated with a decline in performance. Indeed, Lewis et al. (2008) observed improvement in WM performance in the presence of mild acute stressors.

The experimental studies cited above investigated the role of acute stress, but research addressing the cognitive implications of self-reported daily stress has primarily reported negative effects. Wu and Yan (2017) state that chronic stress may negatively affect neuroplasticity and learning. Sliwinski et al. (2006) studied within-person variability of everyday stressors (as opposed to major stressful life events, see Klein and Boals, 2001) and their effect on cognition in young and older adults on six separate occasions. Daily stress predicted variability in response times on a WM updating task in both groups, while only the older group showed negative effects of heightened stress on an attention task. Moreover, stress affected only the more difficult and demanding attention task variant. These findings support the attention depletion hypothesis, suggesting that even everyday stressors may decrease WM and attentional resources. Stawski et al. (2006) conducted a similar study with older adults, arguing that stress impaired cognition through intrusive thoughts and avoidant thinking that appear in response to stressful situations. In young adults, decreased performance on WM updating has been related to negative affect, motivational problems, and reduced attentional control, which are key features in experiencing anxiety or negative stress (Brose et al., 2012). Similarly, work-related stress negatively affected cognitive performance in a sample of Latino workers (Nguyen et al., 2012). A cohort study by Tun et al. (2013) revealed that social strain had the greatest effect on the cognitive performance of those who had low baseline cognitive abilities. Petrac et al. (2009) reported a moderate positive correlation between everyday stress and error rates on attention tasks (both auditory and visual) in undergraduate students, but also a negative correlation between state anxiety and error rates. Thus, while even mild everyday stressors can have an impact on cognitive functioning, there seem to be moderating factors that we are only beginning to understand.

### Aims of the Present Study

The short literature review above indicates that WM performance can be sensitive to stress- or anxiety-related interference. These effects have been extensively studied in clinical and older adult populations. However, less is known about the effects of stress and anxiety on WM in non-depressed adult populations. This lack of research is baffling given the increasing prevalence of stress in a working age population (Wiegner et al., 2015). Experiencing stress and feelings of anxiety are common in otherwise healthy populations, but we know very little about how these mental states are associated with cognitive performance. Many previous studies are also hampered by the fact that they have used only single WM measures (e.g., Moran, 2016). Therefore, the present exploratory study investigated the relationships between WM performance and stress and state anxiety in a large non-depressed adult sample by using questionnaires and an extensive WM test battery including both verbal and visuospatial task variants.

## Materials and Methods

### Participants

Our adult US American participants were recruited online via the Amazon Mechanical Turk (MTurk) crowdsourcing site, and the sample was a sub-set of the sample in the study by Waris et al. (2017); see that paper for more details on recruitment. The participants were selected on the basis of their previous MTurk ratings (95% work approval rating or higher, see also Peer et al., 2014) and the number of completed tasks (more than 100, but less than 1000 task assignments to avoid both inexperienced and very experienced MTurk users). They were also asked whether they had previously participated in similar studies, and 83.9% reported never having done so. Of the 711 participants who completed the study, 159 were excluded due to having more than 10 points on the QIDS-SR16 scale, indicating moderate, severe or very severe depressive symptoms (Rush et al., 2003), since our focus lay on people who according to this cutoff did not currently suffer from depression. Thus, the included participants exhibited at most subclinical levels of depressive symptoms. As the STAI-6 and PSS-4 measures we used (see below) are not clinical diagnostic tools, we did not exclude anyone due to high anxiety or stress scores. Furthermore, we excluded 36 people due to having missing values on the tasks, admitting the use of external aids on WM tasks when probed afterwards, and/or having spent over 24 h on completing the study. We also removed participants who were multivariate outliers on WM task performance (*n* = 13) according to Mahalanobis distance [χ^2^ cutoff = 32.909, *df* = 12]. The final sample included 503 participants.

To investigate the representativeness of our sample vis-à-vis the US adult population, we compared the present sample to the 2015 statistics reported by the U.S. Bureau of Labor Statistics (2016), U.S. Census Bureau Database (2016), and U.S. National Institute of Mental Health (2016). In line with previous MTurk studies (Chandler et al., 2014; Paolacci and Chandler, 2014; Arditte et al., 2016; Keith and Harms, 2016), this comparison indicates that our sample was younger, more highly educated, included more females, exhibited a higher unemployment rate, and had an overrepresentation of people of Caucasian and Asian descent while Hispanic and Black Americans were underrepresented (see Table 1). Several of these features are most likely linked to Internet use in general.

**Table 1:** Demographics of the present sample compared with US adult population statistics.

		The present sample (*n* = 503)	US adult population in 2015
Gender		56.5% female	50.8% female
Age in years	18–24	16.3%	12.6%
	25–44	67%	34.19%
	45–64	15.9%	33.93%
	65–	0.8%	19.27%
Education	High school graduate or higher	41.7%	86.3%
	Bachelor’s degree or higher	56.3%	29.3%
Employment	Unemployed	17.3%	4.6%
Race	Hispanic	8%	17.6%
	Black	8.2%	13.3%
	White	84.5%	77.1%
	Asian	7.8%	5.6%
	Native American	2%	1.2%
	Native Hawaiian/Other Pacific Islander	0%	.2%
	Other/biracial	2.2%	2.6%
Health	Anxiety	15.5%	18.1%
	Depression	14.5%	6.7%


*“Anxiety” and “Depression” denote the percentage of people who reported having received a diagnosis of anxiety or depression at some point in their life.*

### Procedure

The study measures consisted of questionnaires assessing anxiety and stress as well as ten WM tests (see below) that were administered online using an in-house developed web-based programmable testing platform. The platform employs a domain-specific programming language, and it allows researchers to create, distribute, and manage psychological experiments. The MTurk users who were willing to participate received a link through which they accessed and completed the experiment on a computer of their choosing. All participants started with the background questionnaire, after which they completed the ten WM tests. The order of the WM tests was randomized for each participant in order to control for possible test order effects. The only exception to this rule was that the forward single span task (SST) was always administered immediately before the respective numerical-verbal or visuospatial backward SST. On average, the participants completed the entire study in 1 h and 34 min.

### Questionnaires for Stress and Anxiety

#### The Short Form Perceived Stress Scale (PSS-4)

The Short Form Perceived Stress Scale (PSS-4) is an abbreviated version of the self-report Perceived Stress Scale (Cohen et al., 1983). It provides the subjective assessment of stressful life events within the previous month. The PSS-4 consists of four items (items 2, 6, 7, 14 from the original questionnaire) in which the frequency of stressful events is rated on a 5-point Likert scale (*never* to *very often*). The stress dimensions measured are unpredictability, uncontrollability, and sense of overload in everyday life. Individual scores are compared to normative values. The complete 14-item scale has higher reliability than the PSS-4 (*r* = 0.85 as compared to *r* = 0.60) (Cohen and Williamson, 1988), but the brevity of PSS-4 makes it an attractive tool for research.

PSS-4 population norms for non-clinical samples have been gathered in the 1983 Harris Poll in the United States (*N* = 2,387) (Cohen and Williamson, 1988) and in the United Kingdom (*N* = 1,484) (Warttig et al., 2013). When comparing our data to the more recent norms established by Warttig et al. (2013), the total PSS-4 score of our sample is very similar (Table 2). Also the internal consistency of the scale in our sample (α = 0.76) was comparable to that reported by Warttig et al. (2013) (α = 0.77).

**Table 2:** Comparison of average PSS-4 scores in the present sample and in the normative sample collected by Warttig et al. (2013).

	The present sample *n* = 503		Warttig et al. (2013) *n* = 1568	
	***M***	***SD***	***M***	***SD***
Q1	1.44	0.98	1.75	1.05
Q2	1.05	0.94	1.54	1.1
Q3	1.51	0.89	1.19	0.98
Q4	1.27	0.99	1.63	0.85
PSS-4 total	5.27	2.9	6.11	3.14


#### The Six-Item Form of the Spielberger State-Trait Anxiety Inventory (STAI-6)

STAI-6 comprises six items from the State scale of the original State-Trait Anxiety Inventory Y form (Spielberger et al., 1983) that had the highest item-remainder correlations (Marteau and Bekker, 1992). STAI-6 includes three anxiety-present and three anxiety-absent items (respectively, *tense, upset, worried* and *calm, relaxed, content*). The items are formed as statements (e.g., *I feel calm, I am tense*) and each of them is rated on a four-point Likert scale (*not at all* to *very much*). On average, our participants received 10.7 points total in STAI-6. Marteau and Bekker (1992) reported that Cronbach’s alpha of the six-item scale was α = 0.82, as compared to an internal reliability coefficient of α = 0.91 for the 20-item STAI. A later study reported a reliability of α = 0.79 in a study of parental dyads (Tluczek et al., 2009). We obtained an alpha of 0.82, the same as in Marteau and Bekker’s study.

### The WM Measures

Our WM test battery employed four commonly used task paradigms: simple span tasks (forward and backward), complex span tasks, running memory tasks, and n-back tasks. In all task paradigms, two isomorphic variants were administered, namely numerical-verbal (with digits 1–9 as stimuli) and visuospatial (with spatial locations in a 3 × 3 grid as stimuli). Test scores were calculated separately for each test and test variant. Brief descriptions of each task are given below.

#### Simple Span Tasks

In the simple span tasks, the participants were shown stimulus item lists of unpredictable length. In the forward span tasks, the participants reported the presented items in the exact order of appearance, while in the backward span tasks they listed the stimuli in the reverse order. Each test included three- and four-item practice sequences that were administered prior to the task. The proper task involved seven trials comprising stimulus lists that ranged from three to nine items.

The lists were pseudo-randomly generated. All participants were presented with the same set of lists, but the order was randomized. Each item was presented for 1000 ms. In the two verbal versions, an asterisk appeared for 500 ms between digits. In the two spatial versions, the matrix was empty for 500 ms before a new item appeared. There was no time limit set on list recall. The dependent measure was the total number of correctly recalled items, regardless of span length. It was calculated separately for each of the four simple span tasks.

#### Complex Span Tasks

**FIGURE 1:**
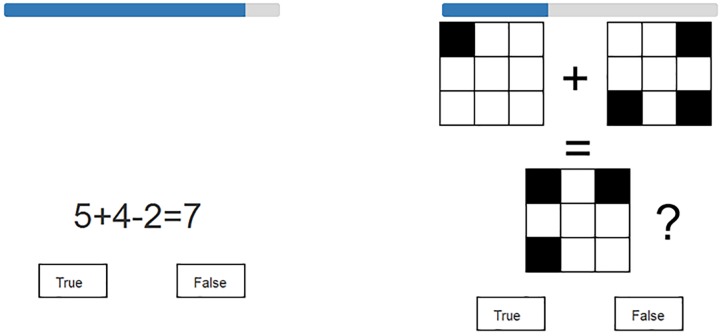
Examples of distractor items in the complex span tasks. Numerical-verbal example item on the left, visuospatial on the right. A timer bar above each item depicts the remaining response time.

Similarly to the forward simple span tasks, in the complex span tasks the participants were presented with stimulus item lists of unpredictable length, and these lists were to be recalled in the exactly same order. However, after each item, the participants had to make a true/false judgment on a distractor item (see Figure 1). In the verbal version, the distractors consisted of arithmetic problems. In the visuospatial version, the participants had to combine two 3 × 3 matrix patterns in their mind and decide whether this combination corresponded to a third pattern. There was a six-second time limit on solving each distractor item.

All participants were presented with the same set of lists, but the order of presentation was randomized. The task comprised five trials consisting of sequences of three to seven items. As in the simple span tasks, item lists were pseudo-randomly generated. Prior to the to-be-remembered item, a fixation point appeared on-screen for 500 ms, followed by the item (1000 ms), fixation point (500 ms) and distractor item (up to 6000 ms). The tests were preceded by a training sequence of three-item and four-item lists. The dependent measure was the total number of correctly recalled items, regardless of span length.

#### Running Memory Tasks

In the running memory tasks, stimulus item lists of unpredictable length were shown. After each list, the participants were asked to report the four last items in the order of presentation. The actual test included eight lists (containing 4–11 items) that were preceded by a practice session.

As in the previous tasks, the stimulus lists were pseudorandomized. Each item appeared on-screen for 1000 ms. In the verbal version, list items were separated by a fixation point (asterisk) that was visible for 500 ms, while in the visuospatial version, the matrix remained empty for 500 ms between items. The number of correctly reported items was used as the dependent measure. The four-item list was excluded from the analyses as it did not require any updating.

#### N-Back Tasks

In the n-back tasks, the participants had to decide for each item whether or not it was the same (target) or not (non-target) as the *n*th item back. In this study, 2-back versions of the task were used. Before each task, the participants had to complete a corresponding practice block. Item lists in the tasks were pseudorandomized, and they included 16 target items, 16 standard non-target items and 16 so-called lure items. The lures were non-targets that would have been targets for the adjacent n-back levels (1-back or 3-back). The potentially distracting lure items were included to avoid test performances that would be based merely on item familiarity.

Each item was presented for 1500 ms. In the verbal version, items were separated by an asterisk presented for 450 ms, and in the visuospatial version, the matrix was empty for 450 ms between items. Overall, the participants had 1950 ms to respond to each item. The dependent measure was the proportion of false alarms (“same” response on non-target items) subtracted from the proportion of hits (correct targets) on the 2-back task.

## Results

### Descriptive Data

Raw accuracy scores obtained from the WM tasks were Box-Cox transformed to decrease skewness and thus improve normality (Osborne, 2010). The overall WM results were comparable to those obtained in laboratory-based studies (Engle et al., 1999).

Table 3 depicts mean accuracy measures prior to the Box-Cox transformation. All scores except the n-back scores denote percentage of correct items; for the n-back tasks, we used the corrected recognition score (i.e., we subtracted the proportion of false alarms from the proportion of correctly recalled target items).

**Table 3:** Mean accuracy rates (SD) on the WM tasks.

Task name	Task type	Dependent variable	Mean (SD)
Simple Span task (forward)	verbal	total number of correctly recalled items	73.14 (17.3)
Simple Span task (forward)	visual	total number of correctly recalled items	62.77 (17.3)
Simple Span task (backward)	verbal	total number of correctly recalled items	63.5 (18.37)
Simple Span task (backward)	visual	total number of correctly recalled items	65.84 (19.8)
Complex Span task	verbal	total number of correctly recalled items	71.73 (28.9)
Complex Span task	visual	total number of correctly recalled items	43.45 (30.8)
Running Memory task	verbal	total number of correctly recalled items	71.6 (20.7)
Running Memory task	visual	total number of correctly recalled items	55.8 (23.7)
2-back task	verbal	proportion of hits minus the proportion of false alarms	59.1 (25.7)
2-back task	visual	proportion of hits minus the proportion of false alarms	53.8 (30.4)


In the subsequent analyses, we employed composite WM variables as they represent more reliable measures than single task scores. Correlations between single tasks are depicted in Supplementary Table S1. Our WM composites were derived from the exploratory factor analysis performed by Waris et al. (2017). We used a data-driven approach rather than *a priori* categorization of the WM tasks to come up with the composites. The reason for this is that previous factor analytic studies on WM tasks have yielded variable results (Waris et al., 2017), and the outcomes of such analyses depend on the particular constellation of tasks used. The exploratory factor analysis by Waris et al. yielded two alternative factor solutions that provided the best fit for the present data: a two-factor model (numerical-verbal factor; visuospatial + n-back factor) and a three-factor model (numerical-verbal factor; visuospatial factor; n-back factor). To retain content-specificity (verbal/visuospatial) that has been considered as the main dimension in the mental organization of WM (e.g., Nee et al., 2012), we used the three-factor solution. Thus, we compiled composite scores for the three latent factors using *z*-transformed task scores. Table 4 shows the bivariate correlations between scores on stress, anxiety and the three WM composites.

**Table 4:** Correlations (Pearson’s *r*) between STAI-6 (anxiety), PSS-4 (stress), verbal WM composite, visuospatial WM composite, and n-back composite (*N* = 503).

	1	2	3	4	5
STAI-6	–				
PSS-4	0.499^∗∗^	–			
Verbal WM	–0.08	–0.05	–		
Visuospatial WM	–0.134^∗∗^	–0.135^∗∗^	0.557^∗∗^	–	
N-back	–0.06	–0.045	0.415^∗∗^	0.558^∗∗^	–


*^∗∗^*p* < 0.01.*

### Factor Analyses of the Stress and Anxiety Measures

Following the factor analyses on the WM tasks, we also conducted separate exploratory factor analyses on PSS-4 and STAI-6. Factorability for both scales was adequate. For PSS-4, the Kaiser-Meyer-Olkin measure of sampling adequacy was 0.74, Bartlett’s test of sphericity was significant [χ^2^(6, *N* = 503) = 487.37, *p* < .001], and the diagonal values of the anti-image correlation matrix were in the 0.72–0.77 range. The exploratory factor analysis on PSS-4 using principal axis factor extraction method with oblique Promax rotation yielded a one-factor solution. The one-factor model accounted for 58,22% of the variance. The model is summarized in Table 5 below.

**Table 5:** Factor loadings on the single-factor model of PSS-4.

PSS-4 question	Factor 1
1	0.68
2	0.60
3	0.65
4	0.73


For STAI-6, the Kaiser-Meyer-Olkin measure of sampling adequacy was 0.79, Bartlett’s test of sphericity was significant [χ^2^(15, *N* = 503) = 1165.4, *p* < 0.001], and the diagonal values of anti-image correlation matrix were in the 0.78–0.83 range. On the basis of principal axis factoring with oblique Promax rotation, a two-factor solution accounting for 72% of the variance was chosen for STAI-6. This model that is summarized in Table 6 shows that anxiety-absent items (*I am calm, relaxed, content*) loaded on factor 1, while anxiety-present items (*I am tense, upset, worried*) loaded on factor 2. As expected, these two factors, mirroring each other through presence vs. absence of anxiety, correlated quite strongly (Pearson’s *r* = 0.57). Moreover, an analysis of bivariate correlations showed similar negative and small associations between the two factors and WM measures (range –0.12 to –0.04). Based on these findings, we decided to employ a single summative score for STAI-6 in the subsequent analyses. It should also be noted that there were no conceptual grounds for the present two-factor solution that might instead be linked to the formulation of the questions and the fact that anxiety-absent items seem to be more strongly correlated with the scale overall (Marteau and Bekker, 1992).

**Table 6:** Factor matrix and factor correlation for STAI-6.

STAI-6 question	Factor 1	Factor 2	Communality
1	0.65	0.18	0.58
2	0.24	0.58	0.55
3	–0.12	0.75	0.48
4	1.01	–0.13	0.88
5	0.63	0.02	0.41
6	0.03	0.78	0.64
Factor 1	–		
Factor 2	0.57	–	


### Linear Mixed Models (LME)

For the statistical analyses, we used the lme4 package (Bates et al., 2015) to compare three models: the null model (including only participant random effects), Model 1 (including the three background variables: age, education, and childhood SES, as well as participant random effects) and Model 2, which also included the variables of interest (PSS-4 and STAI-6 scores). This choice of method allowed us to examine the specific interactions between the variables of interest and WM domains. We decided to keep the background variables in Model 2, as they are known to interact with WM, and because this interaction may differ between WM domains (Myerson et al., 1999; Reuter-Lorenz et al., 2000; Evans and Schamberg, 2009). The formulas of the three models are as follows:

*Y* = *f*(*e*)

*Y* = *f*((age, education, childhood SES) ^∗^ domain) + *e*

*Y* = *f*((age, education, childhood SES, anxiety, stress) ^∗^ domain) + *e*

Here *Y* is the WM performance score, domain is the WM domain (verbal, visuospatial, or n-back), and *e* is the participant random effect. Since WM domain is a categorical variable, we used a deviation coding scheme, in which the mean of each WM domain was compared to the grand mean of overall WM performance. A likelihood ratio test showed that Model 2 was a better fit for the data than the null model [χ^2^(17) = 48.36, *p* < 0.001], or Model 1 [χ^2^(6) = 17.73, *p* = 0.007]. Marginal *R*^2^_GLMM_ for Model 1 was 0.028, meaning that this model explained 2.8% of the variance. Marginal *R*^2^_GLMM_ for Model 2 was 0.045 (Nakagawa and Schielzeth, 2013).

A closer look at Model 2 revealed statistically significant main effects of age, education and domain as well as a trend toward a main effect of anxiety. The model is summarized in Table 7, and regression plots with age and anxiety as predictors are shown in Supplementary Figures S1, S2. We calculated *p*-values using the lmerTest package (Kuznetsova et al., 2017).

**Table 7:** Summary of Model 2 (*N* = 503).

Factor	*df*	Sum of squares	Mean square	*F*	*p*
WM domain	1006	2.28	1.14	3.62^∗^	0.03
Age	503	6.412	6.42	20.4^∗∗∗^	<0.001
Education	503	1.36	1.36	4.3^∗^	0.04
Childhood SES	503	0.12	0.12	0.39	0.53
Anxiety	503	1.12	1.12	3.56.	0.06
Stress	503	0.77	0.77	2.44	0.12
Age ^∗^ WM domain	1006	3.16	1.58	5.02^∗∗^	0.005
Education ^∗^ WM domain	1006	0.66	0.33	1.05	0.35
Childhood SES ^∗^ WM domain	1006	0.12	0.06	0.19	0.85
Anxiety ^∗^ WM domain	1006	0.14	0.07	0.23	0.78
Stress ^∗^ WM domain	1006	1	0.5	1.58	0.2


*^∗∗∗^*p* < 0.001; ^∗∗^*p* < 0.01; ^∗^*p* < 0.05; *p* < 0.1.*

Participants who were younger tended to score higher on the WM tasks. Those who reported a higher level of education also scored higher on the tasks. The main effect of domain was driven by the fact that the mean scores in verbal WM were lower than in visuospatial WM (*z* = 2.63, *p* = 0.02) and in n-back (*z* = 2.15, *p* = 0.06). With regard to anxiety, participants who obtained higher STAI-6 scores also performed worse on the WM tasks (zero-order correlations between WM performance, age, and anxiety scores are presented in Supplementary Figures S1, S2). It is, however, important to note that there was only a trend toward an effect of anxiety, and the WM domain did not interact with anxiety. We also observed an age^∗^domain interaction, as higher age was related to lower performance on visuospatial WM and n-back tasks, but not on verbal WM tasks.

## Discussion

In the present study, we examined how anxiety and stress were associated with WM performance in a large, non-depressed adult sample. Using linear mixed models, we tested the predictive power of the self-report measures of these two factors, as well as background factors (age, education, childhood SES) on WM performance in three different domains (verbal, visuospatial, and n-back). Our analyses revealed main effects of age and education. We also observed a trend toward a main effect of anxiety. Moreover, we found an interaction between WM domain and age: visuospatial WM and n-back performance were negatively associated with age, while verbal WM performance was not. We did not observe significant relationships between stress and the WM measures.

As regards anxiety, our findings are in line with Moran’s (2016) meta-analysis that indicates that increased anxiety (both state and trait) is related to worse WM performance across task paradigms and contents. Our results show that anxiety correlated negatively with both verbal and visuospatial WM performance as well as with n-back task performance, which Moran (2016) calls the dynamic span measure.

We did not find a relationship between stress and WM performance. Our stress measure, PSS-4, focuses on stressful life events experienced during the past month, instead of acute stress linked to the testing situation. In previous research, chronic stress has been reported to show primarily negative effects on cognition (Sliwinski et al., 2006; Stawski et al., 2006). As noted in the Introduction, stress and anxiety are partly overlapping constructs, which is also reflected in the notable correlation between these measures (see Table 4). Hence, it is possible that the STAI-6 score, which we operationalized as transient anxiety, also encompassed feelings of stress.

As regards the limitations of the present study, one concern often discussed in the context of online studies is data quality, as the researcher cannot observe participants’ behavior and performance during the study. However, empirical research on Internet-based cognitive studies shows that their results are comparable to those obtained in traditional experiments, offering good data quality and greater diversity than studies conducted on college samples (Berinsky et al., 2012; Casler et al., 2013; Crump et al., 2013; Goodman et al., 2013; Shapiro et al., 2013; Paolacci and Chandler, 2014). We were careful in taking into account our MTurk participants’ level of experience, quality of previous work, and possible cheating. These control procedures should help to counter several potential pitfalls in Internet data collection. At the same time, recruitment of participants from diverse backgrounds, such as in MTurk, contributes to a greater representativeness of the obtained results. Secondly, there are some possible limitations stemming from our choice of methods. Due to the correlative nature of our data, we cannot make any causal inferences about the interactions between WM, stress, and anxiety. Nevertheless, as our approach allowed the participants to estimate their subjective experience, it can be considered as ecologically more valid than laboratory-induced stress and anxiety. Finally, the questionnaires that we used to measure subjective experience of stress and anxiety have operated on varying time scales: while the PSS-4 asks the participant to assess stress experienced during the previous month, STAI-6 addresses the current mental state. This limits the comparisons between the two measures. Furthermore, one significant limitation of our study is the use of STAI-6 instead of more nuanced measures that could discriminate between components of anxiety (such as worry and arousal). However, we chose the questionnaires on the grounds that they are validated, commonly used, and possess good internal consistency and sufficient discriminatory power despite their brevity.

Future research would benefit from using more detailed measures of anxiety and stress. Another aspect important to address is using measures that operate on comparable time spans. Furthermore, older samples as well as longitudinal studies would also shed some light on the interaction and fluctuation of cognitive performance with anxiety and stress.

In summary, our results showed only a trend toward a negative association between transient anxiety and WM performance. Thus, even demanding WM performance appears to be rather robust against normal variation in everyday stress and anxiety. These findings are relevant for research on cognition-emotion interfaces as well as for testing practices.

## Ethics Statement

This study was carried out in accordance with the recommendations of the Ethics Board of the Åbo Akademi University with written informed consent from all subjects. All subjects gave written informed consent in accordance with the Declaration of Helsinki. Participation was anonymous and all participants were informed of their right to stop at any time. The protocol was approved by the Joint Ethics Committee at the Departments of Psychology and Logopedics, Åbo Akademi University.

## Author Contributions

OW, AS, MiL, and MaL conceived and designed the research. OW aggregated the data. KML, OW, and MiL analyzed the data. KML wrote the original draft. All authors provided critical revisions and approved the final version of the manuscript for submission.

## Conflict of Interest Statement

The authors declare that the research was conducted in the absence of any commercial or financial relationships that could be construed as a potential conflict of interest.

## Supplementary Material

FIGURE S1 Regression plots from Model 1 with age as the predictor (*x*-axis) and the working memory composite scores (n-back, verbal, visual; *y*-axis) as dependent measures.

FIGURE S2 Regression plots from Model 2 with STAI-6 summative score as the predictor (*x*-axis) and the working memory composite scores (n-back, verbal, visual; *y*-axis) as dependent measures.

TABLE S1 Zero-order correlations between the WM tasks.
