# Supplementary figures and images for: The Relationship of Anxiety and Stress With Working Memory Performance in a Large Non-depressed Sample
Source: Front Psychol. 2019 Jan 23;10:4. doi: 10.3389/fpsyg.2019.00004 (PMC6351483; doi:10.3389/fpsyg.2019.00004)

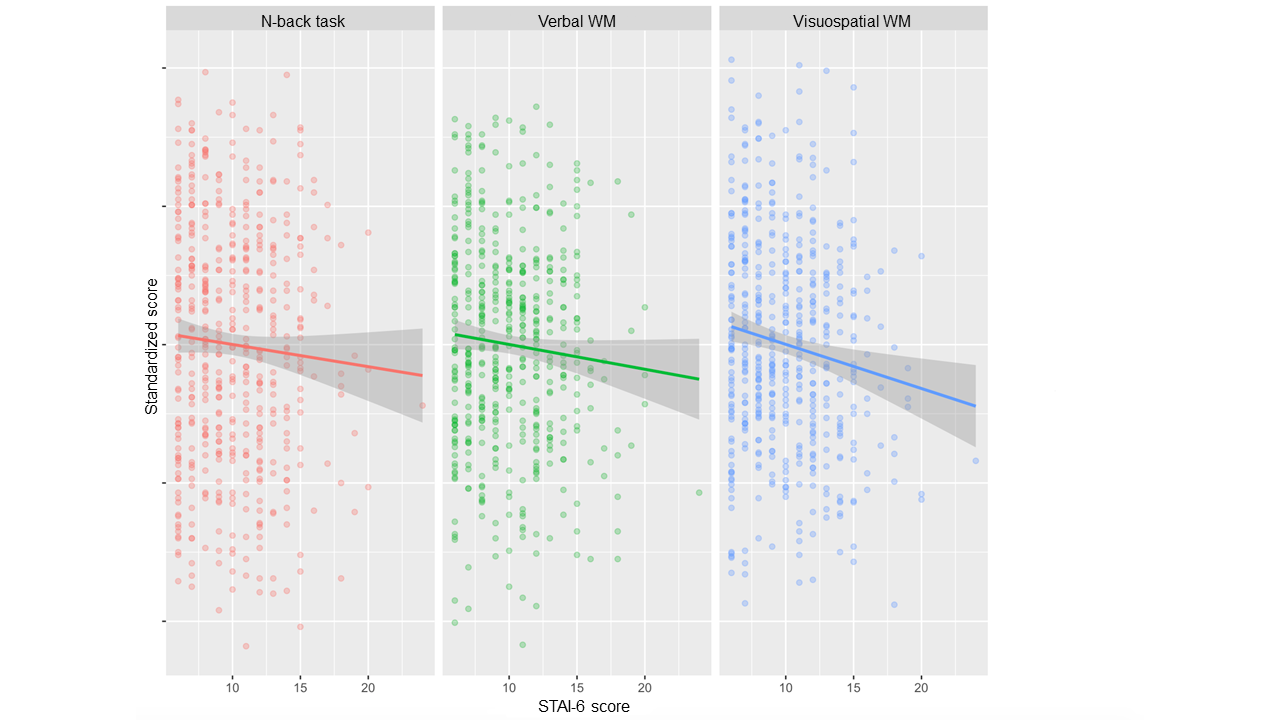

Supplement: FIGURE S1 — Regression plots from Model 1 with age as the predictor (x-axis) and the working memory composite scores (n-back, verbal, visual; y-axis) as dependent measures. [file Image_1.png]

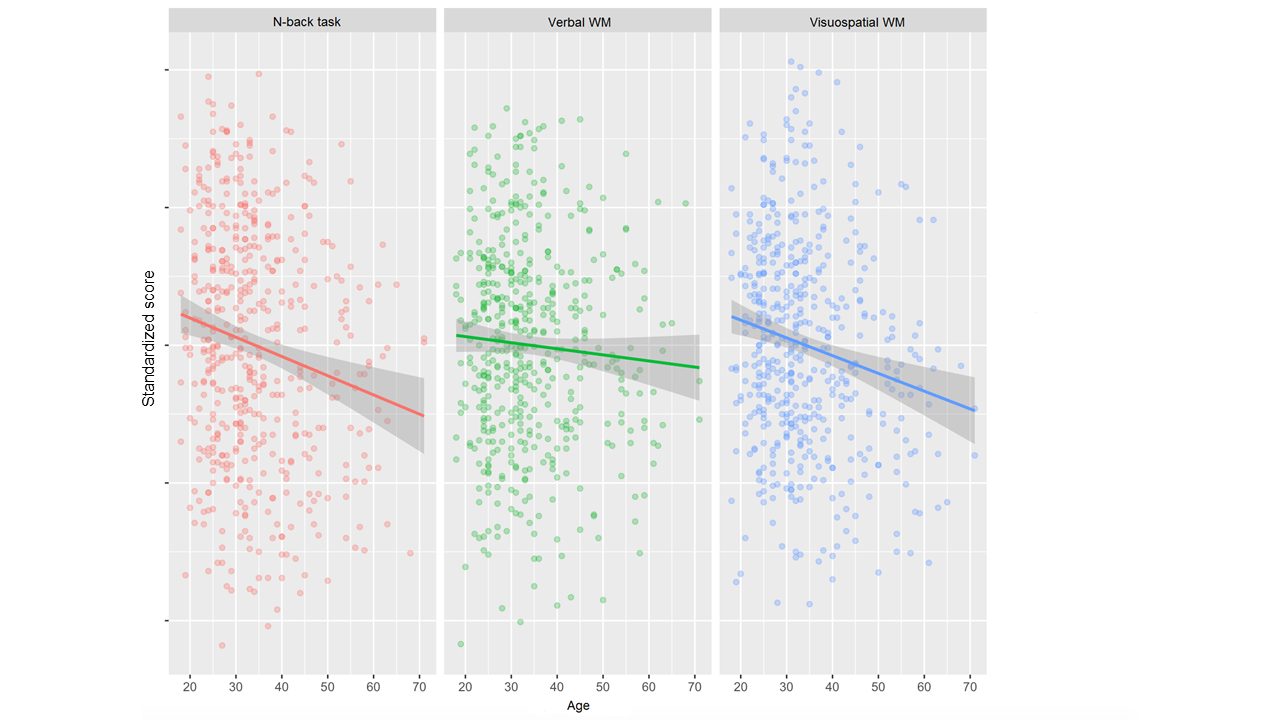

Supplement: FIGURE S2 — Regression plots from Model 2 with STAI-6 summative score as the predictor (x-axis) and the working memory composite scores (n-back, verbal, visual; y-axis) as dependent measures. [file Image_2.png]
