# Supplementary material for: The Relationship of Anxiety and Stress With Working Memory Performance in a Large Non-depressed Sample
Source: Front Psychol. 2019 Jan 23;10:4. doi: 10.3389/fpsyg.2019.00004 (PMC6351483; doi:10.3389/fpsyg.2019.00004)
Supplement: TABLE S1 — Zero-order correlations between the WM tasks. [file Table_1.docx]

Supplementary Table 1

*Zero-order correlations between the WM tasks*

|  | 1 | 2 | 3 | 4 | 5 | 6 | 7 | 8 | 9 | 10 |
| --- | --- | --- | --- | --- | --- | --- | --- | --- | --- | --- |
| 1. Verbal RMT | 1 |  |  |  |  |  |  |  |  |  |
| 1. Visual RMT | .308** | 1 |  |  |  |  |  |  |  |  |
| 1. Verbal CST | .293^**^ | .319^**^ | 1 |  |  |  |  |  |  |  |
| 1. Visual CST | .281^**^ | .436^**^ | .342^**^ | 1 |  |  |  |  |  |  |
| 1. Verbal SSTF | .359^**^ | .291^**^ | .439^**^ | .330^**^ | 1 |  |  |  |  |  |
| 1. Visual SSTF | .271^**^ | .469^**^ | .288** | .489^**^ | .369^**^ | 1 |  |  |  |  |
| 1. Verbal SSTB | .436^**^ | .318^**^ | .450^**^ | .391^**^ | .599^**^ | .350^**^ | 1 |  |  |  |
| 1. Visual SSTB | .283^**^ | .435^**^ | .364^**^ | .407^**^ | .331^**^ | .558^**^ | .376^**^ | 1 |  |  |
| 1. Verbal 2-back | .229^**^ | .370^**^ | .288^**^ | .330^**^ | .250^**^ | .366^**^ | .277^**^ | .435^**^ | 1 |  |
| 1. Visual 2-back | .256^**^ | .396^**^ | .360^**^ | .387^**^ | .238^**^ | .344^**^ | .306^**^ | .409^**^ | .543^**^ | 1 |

Note: double asterisk (**) indicates p < .01.

RMT: Running Memory Task; CST: Complex Span Task; SSTF: Simple Span Task Forward; SSTB: Simple Span Task Backward
